# Regulation of Virulence Gene Expression Resulting from *Streptococcus pneumoniae* and Nontypeable *Haemophilus influenzae* Interactions in Chronic Disease

**DOI:** 10.1371/journal.pone.0028523

**Published:** 2011-12-05

**Authors:** Emily K. Cope, Natalia Goldstein-Daruech, Jennifer M. Kofonow, Lanette Christensen, Bridget McDermott, Fernando Monroy, James N. Palmer, Alexander G. Chiu, Mark E. Shirtliff, Noam A. Cohen, Jeff G. Leid

**Affiliations:** 1 Department of Biological Sciences, Northern Arizona University, Flagstaff, Arizona, United States of America; 2 Department of Otorhinolaryngology–Head and Neck Surgery, University of Pennsylvania, Philadelphia, Pennsylvania, United States of America; 3 Philadelphia Veterans Affairs Medical Center, Surgical Service, University of Pennsylvania, Philadelphia, Pennsylvania, United States of America; 4 Department of Microbial Pathogenesis, Dental School, University of Maryland–Baltimore, Baltimore, Maryland, United States of America; Instituto Butantan, Brazil

## Abstract

Chronic rhinosinusitis (CRS) is a common inflammatory disease of the sinonasal cavity mediated, in part, by polymicrobial communities of bacteria. Recent molecular studies have confirmed the importance of *Streptococcus pneumoniae* and nontypeable *Haemophilus influenzae* (NTHi) in CRS. Here, we hypothesize that interaction between *S. pneumoniae* and NTHi mixed-species communities cause a change in bacterial virulence gene expression. We examined CRS as a model human disease to validate these polymicrobial interactions. Clinical strains of *S. pneumoniae* and NTHi were grown in mono- and co-culture in a standard biofilm assay. Reverse transcriptase real-time PCR (RTqPCR) was used to measure gene expression of key virulence factors. To validate these results, we investigated the presence of the bacterial RNA transcripts in excised human tissue from patients with CRS. Consequences of physical or chemical interactions between microbes were also investigated. Transcription of NTHi type IV pili was only expressed in co-culture *in vitro*, and expression could be detected *ex vivo* in diseased tissue. *S. pneumoniae* pyruvate oxidase was up-regulated in co-culture, while pneumolysin and pneumococcal adherence factor A were down-regulated. These results were confirmed in excised human CRS tissue. Gene expression was differentially regulated by physical contact and secreted factors. Overall, these data suggest that interactions between *H. influenzae* and *S. pneumoniae* involve physical and chemical mechanisms that influence virulence gene expression of mixed-species biofilm communities present in chronically diseased human tissue. These results extend previous studies of population-level virulence and provide novel insight into the importance of *S. pneumoniae* and NTHi in CRS.

## Introduction

The current paradigm of one organism causing disease has been valuable in treating acute infections, however, it has become clear that many chronic bacterial infections are comprised of mixed-species microbial communities. In a healthy state, microbial species exist interdependently among each other and with the host. However, this balance is delicate, and a shift in relative abundances within these complex communities can lead to chronic infection [1]–[6]. Jenkinson and Lamont first described the idea of ‘community virulence’ in the context of oral microbial communities [5]. This paper introduced the idea that interactions among specific microbial communities produce increased or attenuated virulence profiles. In accord with this observation, Ehrlich and colleagues established ‘bacterial plurality’ as a new rubric for understanding chronic infections. Bacterial plurality asserts that bacteria within a population display multiple phenotypes and have multiple genotypes (supragenome), which arise by horizontal gene transfer [1]–[3], [7]. Often, communities of bacteria acting as multicellular organisms express virulence factors that planktonic bacteria do not [3]. Population-level virulence is important in chronic infections including cystic fibrosis, dental caries, otitis media and chronic rhinosinusitis [6], [8]–[11]. Here, we examine chronic rhinosinusitis (CRS) as a model human disease to describe polymicrobial interactions.

Chronic rhinosinusitis is a common disease of the sinonasal tract characterized by symptoms of inflammation lasting for >12 weeks. Several studies have demonstrated the polymicrobial nature of CRS using 454 sequencing and 16S fluorescence in situ hybridization (FISH) [12]–[15]. Recent molecular studies of CRS have confirmed the importance of the respiratory pathogens *Streptococcus pneumoniae* and *Haemophilus influenzae* [12], [16]–[18]. *S. pneumoniae* is a member of the normal flora of the nasopharynx in up to 60% of all healthy children, and carriage rates can approach 100% in some developing countries [4], [19]. Nevertheless, *S. pneumoniae* is implicated in otitis media, pneumonia, and meningitis. Non-typeable *H. influenzae* (NTHi) are nonencapsulated strains found naturally in the human respiratory tract [20]. NTHi is implicated in several diseases including otitis media (chronic and acute) and chronic rhinosinusitis (for a comprehensive review of NTHi see reference [21]). Since *S. pneumoniae* and *H. influenzae* are implicated in otorhinolaryngologic disease, an increased understanding of their interspecies interactions is important.

*S. pneumoniae* has several virulence factors that regulate nasopharyngeal colonization and host tissue damage. Pyruvate oxidase (*spxB*) metabolism results in H_2_O_2_ production, which aids in colonization of the host and confers a competitive advantage with respect to NTHi when establishing microbial communities [22], [23]. Pneumolysin (*ply*) is a hemolytic pore-forming toxin that is important in respiratory infection and host cell damage [22], [24]. Pneumococcal adherence factor A (pavA) binds host fibronectin and is important for bacterial adherence in the upper airway and damaged host tissue [25], [26]. Although not explored in this study, the pneumococcal capsule is an important virulence determinant and can now be differentiated into over 90 serotypes, which can indicate invasiveness and virulence of *S. pneumoniae* [27], [28]. NTHi type IV pili (*pilA*) are important for nasopharyngeal colonization *in vivo* [29], [30]. Type IV pili are involved in biofilm formation and allow NTHi to establish itself within the host resulting in prolonged infection. NTHi biofilms have been implicated in chronic otitis media [31], and recently, chronic rhinosinusitis [16].

In this study, we demonstrate regulation of gene expression between *S. pneumoniae* and NTHi during polymicrobial biofilm growth and confirmed *in vivo* gene expression from excised human CRS tissue. Furthermore, we defined the effects of physical contact and secreted factors on these community interactions. These data support the current hypotheses regarding population-level virulence in chronic polymicrobial disease since virulence factors important for colonization and bacterial persistence were up-regulated in mixed species communities. Since NTHi and *S. pneumoniae* can be cultured from the sinuses using standard clinical techniques, it is relatively easy to identify patients who are co-infected with both organisms. In the future, designing drugs or vaccines that interfere with colonization of the sinuses by *S. pneumoniae* and NTHi will impact the severity of CRS disease by reducing biofilm abundance.

## Methods

### Bacterial growth conditions

Clinical strains of *Streptococcus pneumoniae*, and nontypeable *Haemophilus influenzae* were obtained from the respiratory tract of hospitalized patients. *S. pneumoniae* were grown on tryptic soy (TS) agar and in TS broth. *H. influenzae* were grown on chocolate agar (BD) and brain heart infusion broth supplemented with 2% bovine hemoglobin (Sigma Aldrich) and Isovitalix enrichment agent (BD). All cultures were grown at 37°C under ambient oxygen conditions.

### Growth curve of S. pneumoniae and H. influenzae

Mono- or co-cultures were grown for each species of bacteria in appropriate media overnight. Cultures were diluted 1∶100 into fresh media. Optical density (600nm) was recorded on an Eppendorf spectrophotometer. Readings were taken every hour for a total of 10 hours. Bacteria were diluted, plated on chocolate agar and TS agar, and CFU calculated at 0 minutes, 255min, 485min, and 600min after inoculation with overnight cultures of each species.

### Biofilm growth

Biofilms were grown in 24-well polystyrene plates (Falcon) as previously described [32]. For co-cultures, an equal volume of each diluted log-phase culture was added to each well to achieve a total volume of 500 µl. The plates were covered and incubated at 37°C without agitation for 24 hours. Biofilm cells were harvested by sonication for 30 seconds after inverting the plates to remove unattached cells. Biofilm cells were collected in 1.7 mL eppendorf tubes, centrifuged to pellet, and stored in RNAlater for RNA extractions, below.

### RNA extractions for reverse transcriptase

RNA was extracted using a RiboPure-Bacteria kit (Applied Biosystems) per manufacturer protocol. Following extraction, RNA was treated with DNase I (Applied Biosystems) to remove any DNA contaminants. RNA concentration and purity was determined by measuring absorbance at both 260 nm and 280 nm on a NanoDrop ND-2000 spectrophotometer (Thermo Scientific). For cDNA synthesis, RNA was normalized to 1 ng/µl. First strand cDNA was synthesized using a High Capacity cDNA reverse transcription kit (Applied Biosystems) per manufacturer protocol. A negative control containing the reaction components without reverse transcriptase was included to ensure no DNA contamination was present.

### Relative gene expression

Gene expression was determined using a SYBRgreen (Applied Biosystems) real time PCR assay. Gene specific primers were designed in the open reading frame of each gene of interest, Table 1. The 20 µl reactions consisted of 1X SYBRgreen mastermix, 1 µM each primer, and 2 µl of cDNA. Real time cycler conditions were 1X of 50°C for 2 min, 1X of 95°C for 2 min, 40X of 95°C for 15 sec, and 60°C for 1 min, 1X of 72°C for 5m, followed by a melt curve of 71X at 5 degree intervals for 5 seconds each. No reverse transcriptase (NRT), and a no template control (NTC) were included to verify the reaction mixtures were contaminant-free. A positive control of pure DNA from each species was included to ensure the reaction conditions were appropriate and gene amplification occurred during each run. We used the ΔΔCt method to determine relative gene expression of the gene of interest in co-culture v. mono-culture biofilms normalized to expression of the 16S rRNA gene for each organism. This allowed for visualization of up or down regulation of a particular gene when two species were in co-culture. Results are reported as ΔΔCt and graphed appropriately. Fold change was calculated by using the exponential 2^ΔΔCt^. Significance was determined using Student's t-test. A p≤0.05 was considered statistically significant.

**Table 1:** List of primer sequences and target gene function.

Organism	Primer ID	Primer Sequence 5′-3′	Gene Target	Gene Function	Reference/ Accession Number
*Haemophilus influenzae*	HFLU-F	TCCTAAGAAGAGCTCAGAGAT	16s	Ribosomal RNA	[59]
*Haemophilus influenzae*	HFLU-R	TGATCCAACCGCAGGTTCC	16s		
*Haemophilus influenzae*	pilA-F	CTATATACACATAATTCCACATCAGCCTTA	pilA	Colonization, biofilm formation	[30], [60]
*Haemophilus influenzae*	pilA-R	CCACCATCGCAATTCCTTCTT	pilA		
*Streptococcus pneumoniae*	STPN-F	CTGCGTTGTATTAGCTAGTTGGTG	16s	Ribosomal RNA	[61]
*Streptococcus pneumoniae*	STPN-R	TCCGTCCATTGCCGAAGATTC	16s		
*Streptococcus pneumoniae*	spxB-F	ATTCGGCGGCTCAATCGGGG	spxB	Production of H2O2	NC_008533.1
*Streptococcus pneumoniae*	spxB-R	CAGCACGGCAGGCTTCGTCA	spxB		
*Streptococcus pneumoniae*	Ply2-F	CTACCCGATGAGTTTGTTGTT	ply	Cytolysin	[61]
*Streptococcus pneumoniae*	Ply2-R	TCCAGGATAGAGGCGACT	ply		
*Streptococcus pneumoniae*	pavA-F	GGTCGCATCCAGAAAATC	pavA	Adherence and inflammation	[61]
*Streptococcus pneumoniae*	pavA-R	AGAAAGGAGCAGGCGATG	pavA		

Genes for NTHi and *S. pneumoniae* are listed along with primers sequences (5′-3′), gene target, function, and GenBank accession number, if applicable.

### Scanning electron microscopy

NTHi was grown in mono and co-culture for 24h in 4-chamber glass slides. Following incubation, bacteria were fixed with 4% paraformaldehyde for 4h at room temperature. Slides were dried in ethanol, desiccated overnight, and sputtered with gold for SEM. Mono-culture NTHi and co-culture NTHi and *S. pneumoniae* biofilms were observed at 10,000X magnification.

### Ethics Statement

Tissue samples from human sinuses were obtained from the Hospital of the University of Pennsylvania. A total of 10 patients undergoing functional endoscopic sinus surgery (FESS) were solicited. The Hospital of the University of Pennsylvania Institutional Review Board specifically approved this study under the approval number #800614. Written informed consent was provided by study participants.

### RNA isolation from excised human sinus tissue

Following biopsy, tissue was stored in RNAlater and shipped to NAU for analysis. A total of 7 CRS and 3 non-CRS patients were used for these studies. Total bacterial RNA was extracted from the excised human sinus tissue using the RiboPure – Bacteria with the following modifications to manufacturer protocol. Approximately 25 mg of tissue was homogenized using 0.1 mm zirconia beads and one 5mm steel bead on a Qiagen TissueLyser in RNAwiz (Applied Biosystems) at 4^o^C. The homogenate was transferred to a new tube and 0.2 volumes of chloroform added. The RNA mixture was incubated at room temperature for 10 min. Following incubation, the mixture was centrifuged and the top layer containing partially purified RNA removed. Ethanol was added and the mixture placed directly on a spin column provided with the kit. RNA was washed 3X, and eluted. Purified RNA was treated with DNase I and quantified as described above.

### *Ex vivo* gene expression

The presence of bacterial RNA was determined by universal 16S primers in a SYBRgreen assay at a 1 µM concentration. The primers were 1406f, 5′-??? TGYACACACCGCCCGT-3′ (universal, 16S rRNA gene), and 23Sr, 5′-??? GGGTTBCCCCATTCRG-3′ (bacterial-specific, 23S rRNA gene) [33]. Relative bacterial gene expression was determined as described above.

### Physical contact co-culture assay

To determine the role of physical contact in the production of community virulence factors, we utilized 0.22 µM tissue culture inserts as described previously [34]. To ensure biofilm confluence on the tissue culture insert, bacteria were stained with Syto-9 and visualized using fluorescence microscopy (data not shown). Biofilms were incubated for 24 hours and RNA isolated as described above. cDNA was used to assay for gene expression and ΔΔCt are reported below.

### Treatment of biofilm bacteria with supernatant fluid

To investigate whether polymicrobial-specific gene regulation was mediated by secreted factors, co-culture biofilms of *H. influenzae* and *S. pneumonia*e were grown in 24-well polystyrene plates (Falcon) as previously described. After 24 hours, supernatant fluid (SNF) was extracted into sterile 50 mL conical tubes (Fisher Scientific) and centrifuged at 4,000x rpm for 20 minutes to remove cellular components. The SNF was then decanted into a new, sterile 50 mL conical tube and filter sterilized (Millipore, 0.22 µm pore diameter). Mono-culture biofilms were inverted to remove the spent nutrient-rich media and the sterilized, conditioned SNF was then introduced and the biofilms incubated at 37°C for an additional 24 hours. Cells were harvested by sonication for 30 seconds, and RNA was extracted as described above. Complementary DNA was synthesized and used in rtPCR assays for genetic analysis. Controls of nutrient deprived media were employed to rule out nutrient deprivation as a main factor in gene expression changes.

### Statistical methods

For rtPCR analysis, mean Ct values were calculated across trials. Three independent trials were conducted unless otherwise stated. Standard deviation was reported and Student's t-test was used to determine significance compared to control. Significance was determined by comparing raw Ct values to the number of PCR cycles used in each assay. Co-cultures were normalized to mono-culture expression of each gene by the following equation, ΔΔCt  =  ((µCt(mono-culture) – µCt(co-culture)) –(µCt(16s monoculture)- µCt(16s coculture)), where µ is the mean. Fold change was calculated by using the exponential 2 ^ΔΔCt^. Ct differences were considered significant when p≤0.05.

## Results

### Planktonic growth is not inhibited in co-culture conditions

**Figure 1:**
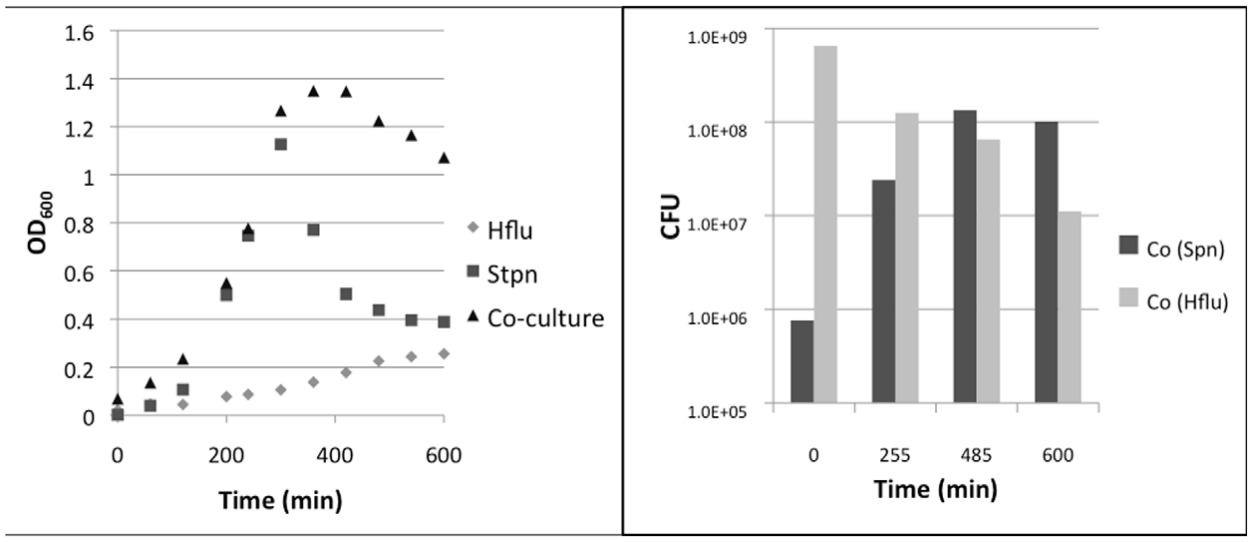
Growth curve and colony forming units of planktonic mono and co-cultures of NTHi and *S. pneumoniae*. Planktonic NTHi and *S. pneumoniae* were grown in sBHI for 10h. Optical density (600nm) and CFU were recorded. Growth inhibition was not detected in co-culture by this method.

To confirm that gene regulation observed in co-culture biofilms was not a product of cellular death or inhibition of growth, we measured the optical density of mono- and co-culture bacteria over 10 hours of planktonic growth. These studies demonstrated that growth inhibition did not occur in co-culture conditions (Figure 1). Conversely, the curve suggested a synergistic interaction in the planktonic state, since co-culture bacteria grew more vigorously. Colony forming units were calculated periodically throughout the growth curve beginning at time 0, directly after inoculation with *S. pneumoniae* and NTHi. Both NTHi and *S. pneumoniae* survived to 10h, well into the stationary phase of growth, indicating that co-existence can occur *in vitro*.

### *H. influenzae* type IV pili is expressed only in co-culture conditions

**Figure 2:**
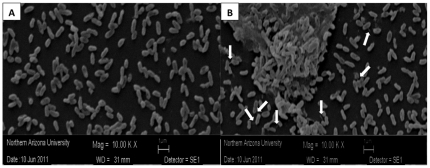
Scanning electron microscopy of NTHi mono-culture and co-culture with *S. pneumoniae* show increase in pili-like structures in co-culture. NTHi was grown for 24h in mono- and co-culture with *S. pneumoniae* and SEM was used to identify pili-like structures. While no pili could be identified at 10,000X magnification in mono-culture, at least 7 structures were identified in co-culture with *S. pneumoniae*.

To investigate gene regulation when *H. influenzae* was cultured with *S. pneumoniae,* the transcript for NTHi type IV pili (*pilA*) and was examined and compared to expression of NTHi 16S rRNA as a reference gene. Type IV pili are important for nasopharyngeal colonization and in vivo growth of *H. influenzae* [30], [35]. Since *pilA* is important for early biofilm formation, mono- and co-culture biofilms were grown for 4h and 24h and relative gene expression was calculated. Following each co-culture experiment, bacteria were plated to ensure growth of both organisms after 4 and 24 hours (data not shown). We found that *pilA* was only expressed in co-culture conditions (Table 2) at both 4h and 24h (p = 0.001, and 0.013, respectively). For this study we examined *S. pneumoniae* and NTHi mediated induction of *pilA*, however, other studies in our lab suggest that this not a species-specific effect (data not shown). Scanning electron images of NTHi mono-culture biofilm (Figure 2, panel A) and co-culture with *S. pneumoniae* (Figure 2, panel B) were also observed at 10,000X to identify pili. There is an increase in pili-like structures from the rod-shaped cells (NTHi) in the co-culture setting, whereas no pili-like structures could be detected in mono-culture after 24h of growth.

**Table 2:** Real time PCR analysis of expression of NTHi type IV pili.

	2^ΔΔCt^ (Fold change from reference gene)	
	4h	24h
*pilA*	9.33E+07*	9.17E+07*
	(+ 1.94)	(+ 2.53)

NTHi were grown in mono- and co-culture biofilms and expression of 16s rRNA reference gene and *pilA* are recorded and the ΔΔCt calculated. Fold change, or 2^ΔΔCt^, from the reference gene are displayed in panel B. Standard deviation is presented, and changes are considered significant when p≤0.05, determined by Students t-test. Significance is indicated by *.

### *S. pneumoniae* virulence genes are differentially regulated in co-culture with *H. influenzae*

**Figure 3:**
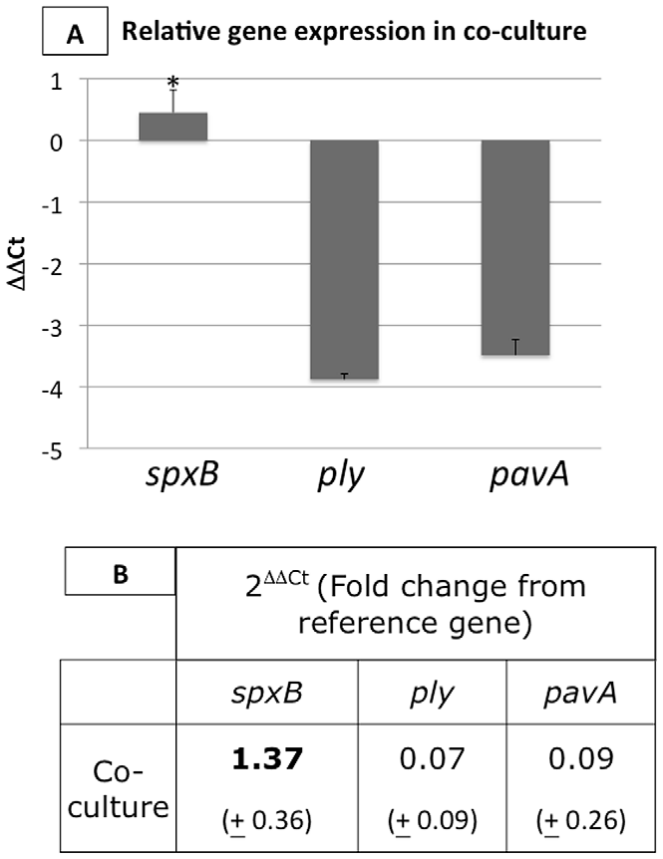
Real time PCR for expression of *S. pneumoniae* housekeeping and virulence genes show differential gene regulation in co-culture with NTHi. *S. pneumoniae* were grown in mono- and co-culture biofilms and expression of *spxB, ply,* and *pavA* are recorded and normalized to expression of the 16s rRNA gene using the ΔΔCt method. Up and down-regulation are displayed as ΔΔCt in panel A. Fold change from the reference gene are displayed in panel B. Changes are considered significant when p≤0.05. Significance is indicated by *. Here, we show significant up-regulation of *spxB* in co-culture with NTHi, and down-regulation of *ply,* and *pavA.*

To further define the products of interactions between *S. pneumoniae* and *H. influenzae*, we examined the transcription of several *S. pneumoniae* virulence factors in mono- and co-culture. Genes of interest included pneumolysin *(ply),* pneumococcal adherence factor A *(pavA)*, and pyruvate oxidase *(spxB)*. These virulence factors are important in nasopharyngeal colonization *in vivo*, and have been implicated in respiratory tract infection [23], [24], [36]. For a reference of normal gene expression, streptococcal 16S rRNA gene was also included. Notably, *spxB* was up-regulated when in co-culture with *H. influenzae* for 24h (Fold change, 1.37, p = 0.001), while the other virulence factors, *ply* and *pavA,* were down-regulated (p = 001, and p = 0.012, respectively). The ΔΔCt values of co-culture compared to mono-culture are demonstrated in Figure 3 panel A, and fold change is described in Figure 3 panel B.

### Virulence gene regulation occurs in complex communities *in vivo*

**Figure 4:**
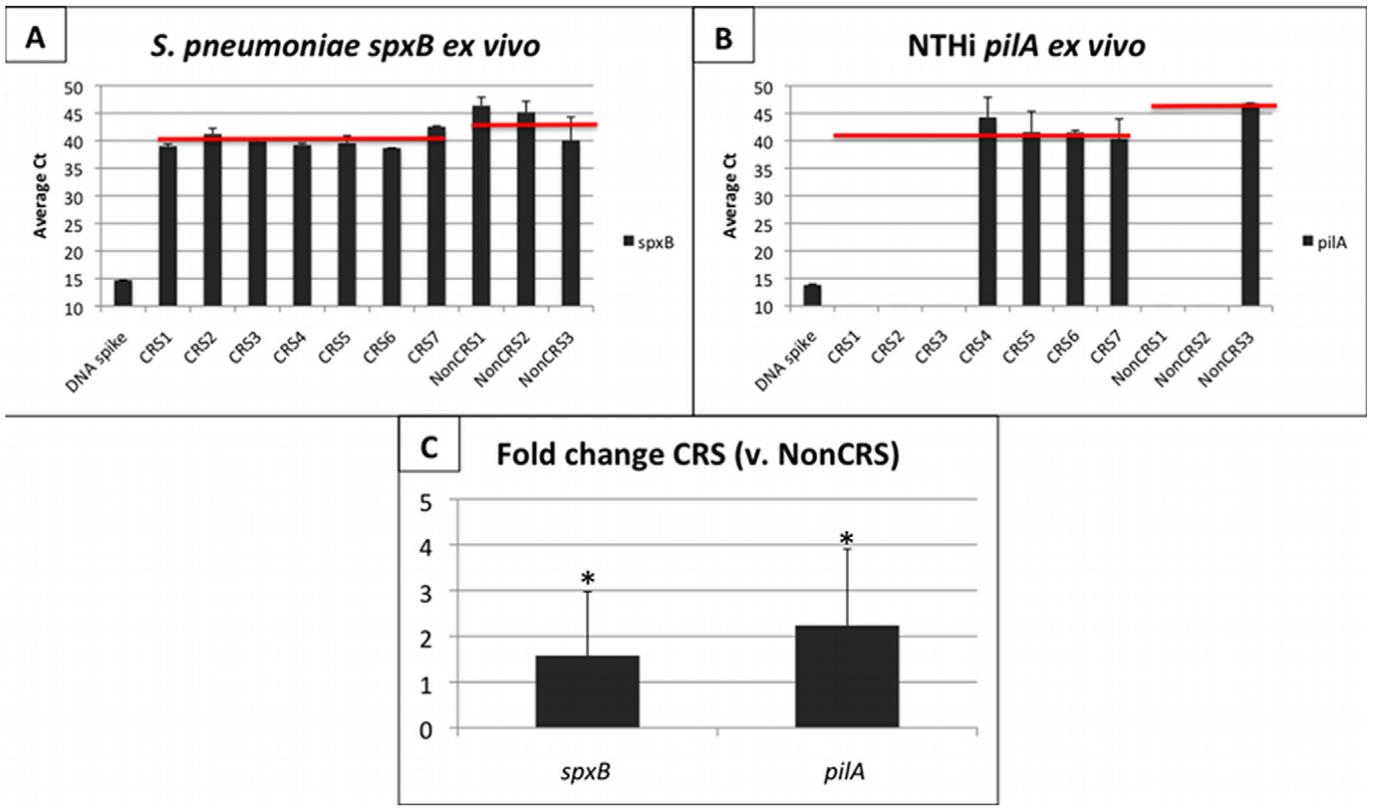
Real time PCR for *S. pneumoniae* and NTHi virulence gene transcripts from CRS and non-CRS excised tissue. Transcripts for virulence factors of *S. pneumoniae* and NTHi were observed in human tissue from 7 CRS and 3 non-CRS patients (x-axis). A positive control of the appropriate DNA spike was included. Panels A and B show the average Ct for *spxB* (A) and *pilA* (B) genes, and the average among all positive samples in CRS and non-CRS (red bar). Panel C displays the fold change (2^ΔΔCt^) of *spxB* and *pilA* relative to the appropriate 16s rRNA gene expression in CRS vs. non-CRS tissue. This figure shows the presence of *S. pneumoniae spxB* in 7/7 CRS and 3/3 non-CRS; however, expression was significantly greater in CRS patients (P = 0.002). NTHi *pilA* was demonstrated in 4/7 CRS samples and 1/3 non-CRS samples. Expression of *pilA* was significantly greater in CRS than non-CRS patients. Standard deviation is presented and significance (P<0.05) is indicated by *, determined by students t-test.

To investigate whether these genes were expressed in diseased human sinus tissue from patients with chronic rhinosinusitis (CRS), we isolated bacterial RNA from excised human sinus tissue. Although the communities present in the sinuses are notably complex [12], we hypothesized that the interactions observed *in vitro* are likely to occur in vivo in a chronic disease setting. Both *pilA* and *spxB* were expressed in CRS associated microbial communities, (Figure 4). Reference 16S genes from each organism were included to calculate ΔΔCt and fold change (Figure 4C). *S. pneumoniae spxB* was present in 7/7 CRS patients and was up-regulated in CRS tissue (average Ct  = 40.02) compared to non-CRS control (average Ct  = 43.87). When normalized to the expression of *S. pneumoniae* 16S rRNA, *spxB* was expressed 1.58x greater in CRS vs. non-CRS patient tissue samples. Expression of *spxB* in important for nasopharyngeal colonization *in vivo*, so expression in samples without NTHi is not surprising, especially in the presence of polymicrobial communities in CRS tissue. NTHi *pilA* was expressed in 4/7 CRS patients, and in 1/3 non-CRS patients. Expression of *pilA* was significantly increased in CRS (average Ct  = 41.93) compared to non-CRS tissue (average Ct  = 46.85, p = 0.003). When normalized to the 16S reference gene expression, *pilA* was expressed 2.24x greater in CRS vs. non-CRS tissue.

### Physical contact regulates *S. pneumoniae* and NTHi gene expression

**Figure 5:**
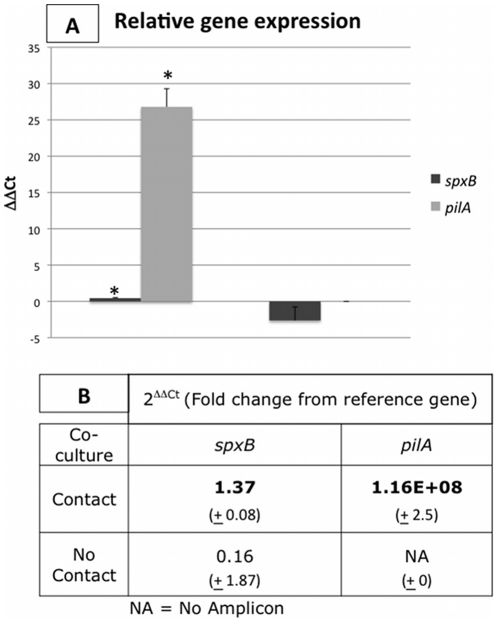
Physical contact regulates the expression of NTHi *pilA,* not *S. pneumoniae spxB.* Virulence factors *pilA* and *spxB* were assayed for when *S. pneumoniae* and NTHi were in mono-culture, co-culture with physical contact (dark gray bars), and in co-culture without physical contact (light gray bars). The ΔΔCt is presented in Panel A. Fold change from 16s gene expression is displayed in Panel B. Expression of *pilA* was induced only when physical contact was allowed. *S. pneumoniae spxB* was only significantly up-regulated when physical contact was allowed (p = 0.002). Standard deviation is presented and significance (P<0.05) is indicated by *, determined by students t-test.

To investigate whether physical contact played a role in virulence gene regulation, we grew co-cultures of both organisms separated by a trans-well tissue culture insert [34]. Genes that were up-regulated in co-culture were included in these studies. When physical contact was allowed, *spxB* was expressed 1.37x greater in co-culture with NTHi (Figure 5). Interestingly, *S. pneumoniae spxB* was slightly down-regulated in the absence of physical contact, although this result was not statistically significant. NTHi expression of *pilA* was induced only when physical contact with *S. pneumoniae* was allowed. No gene expression of *pilA* was observed when NTHi was grown in mono-culture or in the trans-well assay with *S. pneumoniae*.

### Soluble factors in supernatant fluid from co-culture biofilms influence mono-culture gene expression

**Figure 6:**
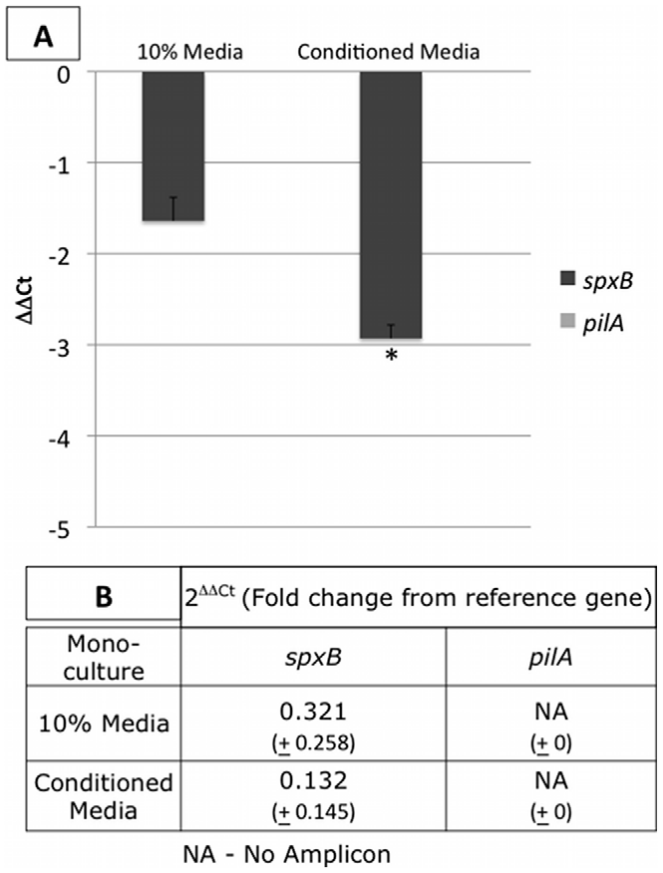
Chemical factors secreted in co-culture conditions regulate the expression of key virulence genes. Conditioned media from *S. pneumoniae/*NTHi 24h co-cultures was used to treat 24h single species biofilms (Conditioned Media column). Cells were grown in 100% media, and treated with 100%, or 10% media to distinguish the effects of nutrient depletion. The ΔΔCt is presented in Panel A. Fold change from 16s gene expression is displayed in Panel B. Conditioned media did not induce *pilA* expression. *S. pneumoniae spxB* and the 16s rRNA genes were down-regulated in the presence of CM compared to the media controls. Student's t-test determined significant down-regulation of *spxB* only when conditioned media was used.

To assess whether soluble factors produced during co-culture growth influenced mono-culture gene expression, we employed a conditioned media assay (Figure 6, experimental details provided above). To ensure that the effects on gene expression were not solely due to nutrient starvation, we included a 10% media control. We found that expression of *pilA* was not induced by soluble factors, suggesting that secreted factors do not regulate type IV expression in NTHi in this assay system. *S. pneumoniae* expression of *spxB* suppressed in the presence of conditioned media, which may indicate a negative interaction on cell growth or metabolic activity when only soluble factors from co-cultures are present. While it is clear that nutrient starvation decreased expression of *spxB,* media conditioned with co-culture soluble factors was significantly different (p = 0.0002), suggesting that some down-regulation can be attributed to secreted factors in the co-culture setting.

## Discussion

In this study, we investigated the regulation of virulence determinants in *S. pneumoniae* and *H. influenzae* clinical isolates as a function of community interactions. While competition between strains of *S. pneumoniae* and NTHi has been observed [37], [38], our analyses did not demonstrate any measurable competition that would compromise these data. Rather, growth curve and CFU analysis of dual NTHi and *S. pneumoniae* cultures suggested an additive effect. These discrepancies could be explained by the use of vastly different strains of NTHi and *S. pneumoniae* between these studies. For example, Weiser and colleagues demonstrated an inverse relationship between *S. pneumoniae* and NTHi colonization *in vitro*, largely due to *S. pneumoniae* production of pyruvate oxidase, using the typed laboratory strain P294 (*S. pneumoniae* serotype 4) [37]. However, the isolates of *S. pneumoniae* and NTHi used in the studies presented here were clinical strains directly from the human nasopharynx. It should be noted that due to the clinical nature of these strains, *S. pneumoniae* was not fully characterized and serotype information was not available. We recognize the enormous intraspecies heterogeneity of *S. pneumoniae*, and appreciate this limitation of these studies. Over 90 pneumococcal serotypes varying in capsular structure have been identified, and associations between serotypes and colonization of the upper respiratory tract have been described [39]. For example, in a large-scale epidemiological study of acute otitis media in Germany, serotypes 19F and 3 were the most commonly isolated [40], while serotypes 1, 5, 6, 14, 19F, and 23F were most commonly isolated from invasive pneumococcal disease [41]. While serotype information would help to clarify disparities in the literature, we feel that the strains used in these studies represent potential *in vivo* interactions.

The frank *S. pneumoniae* virulence factors streptococcal pneumolysin and pneumococcal adherence factor A were down-regulated in co-culture with *H. influenzae.* Streptococcal pneumolysin is a cholesterol dependent cytolysin and is an important virulence determinant of pneumonia [42]. Pneumococcal adherence factor A is required for optimal adherence to host cells and is responsible for increased virulence in a mouse model [25], [26]. These results were expected because these factors are often associated with acute infections in humans. Chronic rhinosinusitis, and other biofilm-mediated diseases, are not characterized by frank epithelial damage from bacterial virulence factors, but by prolonged inflammation caused by persistence of pathogenic microbial communities.

Expression of streptococcal pyruvate oxidase *(spxB)*, an enzyme that is important in the production of hydrogen peroxide, was up-regulated in the co-culture setting. Hydrogen peroxide production has been extensively studied in *S. pneumoniae* as a means for nasal colonization [23], [43]–[45]. Pyruvate oxidase also confers a competitive advantage for nasopharyngeal colonization *in vivo* partly because *S. pneumoniae* is resistant to self-produced H_2_O_2_ killing while other species are susceptible during stages of early colonization [23], [44]. Interestingly, Allegrucci and colleagues demonstrated that maximal production of *spxB* occurs in mature single-species biofilms (6 days) [46]. Our studies show an increase of *spxB* transcription in 24h co-culture biofilms, suggesting that streptococcal virulence is intensified with respect to colonization and competition in co-culture. Other streptococcal virulence factors play a role in nasopharyngeal colonization including the capsule, the adhesion molecule phosphorylcholine (choP), choline-binding proteins, and exoglycosidases [26]. We are currently investigating the role of these genes in streptococcal community virulence *in vitro* and *ex vivo*.

*H. influenzae* virulence factor type IV pili (*pilA*) was expressed only in mixed-species biofilms. Type IV pili are important in biofilm formation, epithelial colonization and colonization of the upper respiratory tract. Prior to the study by Bakaletz and colleagues (2005), NTHi were not considered to produce functional type IV pili [47]. This was, in part, because single species cultures of NTHi *in vitro* do not express important attachment proteins when grown on common growth media. However, Bakaletz *et al.* visualized type IV pili under defined growth conditions. Recently, Jurcisek and colleagues (2007) demonstrated that type IV pili have an important role in biofilm formation and epithelial attachment *in vivo* [29]. Here, we show *pilA* expression is inducible in co-culture *in vitro* and is expressed in chronically diseased sinus tissue. These studies support recent findings by Swords and colleagues that NTHi interacts with pneumococci to promote biofilm formation in a chronic infection [48]. While further study is necessary to define the implications of NTHi *pilA* and *S. pneumoniae spxB* in disease, these data provide an important first step to understanding the mechanisms of polymicrobial persistence in disease. Likely, when in the same niche, *S. pneumoniae* and NTHi aid each other to adhere to the host epithelia and in the eventual establishment of a polymicrobial community that is recalcitrant to antibiotic treatment.

We confirmed the presence of *S. pneumoniae* and NTHi virulence transcripts in excised sinus tissue from 7 CRS and 3 non-CRS patients. Non-CRS patients were selected based on the absence of inflammation in the sinonasal cavity but are not classically ‘healthy’ patients. Nonetheless, transcription of *S. pneumoniae spxB* was increased in CRS compared to non-CRS tissue, regardless of the presence of NTHi. While our *in vitro* assays show that interaction with NTHi increased expression of streptococcal pyruvate oxidase, it is likely that the complex microbial communities in the human sinus also interact to increase gene expression of *S. pneumoniae spxB* (data not shown). Transcription of *H. influenzae pilA* is indicative of physical interactions among multispecies communities of microbes since it was only transcribed in the presence of another species *in vitro*. Importantly, we did not rule out host-pathogen interactions as a contributing factor to increased virulence gene expression in sinus tissue specimens. Recent studies have suggested that metabolic stress, components of the host immune system, and microenvironments within the host can influence bacterial virulence and invasiveness [49]-[52]. CRS is broadly characterized by a common endpoint of sinonasal inflammation. This response is notably complex, and results in an influx of inflammatory cells, effector molecules and small cationic peptides that likely influence bacterial gene expression [53]–[55]. In addition to the presence of polymicrobial communities in the diseased sinus, host-pathogen interactions may account for the increase of *S. pneumoniae spxB* expression even in the absence of NTHi. In future studies, we aim to elucidate the mechanisms that contribute to pathogenesis of *S. pneumoniae* and NTHi in CRS. Additionally, further investigation of the disease state of these patients is underway to see if NTHi with type IV pili and *S. pneumoniae spxB* are correlated with more severe or recalcitrant CRS disease. Importantly, these results confirmed our *in vitro* analysis and demonstrated their clinical relevance in diseased tissue.

Bacteria have several mechanisms to mediate interactions in a community setting. Secreted factors, which may include quorum sensing molecules, secondary metabolites, carbohydrates, or proteins, can influence gene regulation in microbial communities. To test for this interaction, while not ruling out additional factors, we employed a conditioned media assay, described above. To rule out changes in gene expression due to nutrient depletion, we included a depleted (10%) media control. These data clearly show that NTHi *pilA* was not inducible by soluble components alone. Interestingly, *S. pneumoniae spxB* was down-regulated in the presence of conditioned media. This shows that soluble factors alone have a depressive effect on this virulence gene. This may be important when *S. pneumoniae* co-infects with another species, but does not share a niche. In this scenario, pyruvate oxidase metabolism would not be necessary as a competitive factor. *S. pneumoniae* and NTHi have been shown to co-infect, but form separate mono-species biofilms, in a tightly controlled chinchilla model of otitis media [48]. However, it is unlikely that this would occur in a complex chronic infection such as CRS, where there are several co-infecting species in addition to the host's normal flora. Importantly, while we have not completely ruled out the potential importance of secreted factors, we show that these factors alone cannot induce expression.

A second mechanism we examined was the effect of direct physical contact. NTHi *pilA* was expressed when physical contact occurred, suggesting physical interaction was important for expression of type IV pili. When physical contact was blocked, *pilA* expression was not induced. This may be similar to the interaction with host cells in *in vivo* infection models [29]. Since type IV pili are required for twitching motility, colonization, and biofilm formation, understanding these mechanisms may aid in treatment of many otolaryngologic and respiratory diseases. For example, Bakaletz and colleagues have demonstrated that immunization against NTHi type IV pili confers significant protection against NTHi infection in a chinchilla model of otitis media [56]–[58]. Suppression of *spxB* in *S. pneumoniae* when exposed to conditioned media reveal that secreted factors from *H. influenzae* has a negative effect on virulence in *S. pneumoniae*. Additionally, physical contact alone did not regulate *spxB* expression. However, when physical contact and secreted factors were coupled in a standard co-culture assay, maximum expression was observed. These data suggest that interactions between *S. pneumoniae* and NTHi are more complex than a single mechanism and warrant additional studies.

Overall, these data suggest that interactions between *H. influenzae* and *S. pneumoniae* involve physical (adherence) and chemical (secreted) mechanisms that influence virulence gene expression of mixed-species biofilm communities present in chronically diseased human tissue. The findings generated from these studies contribute to the understanding of the complex and clinically significant interactions between *S. pneumoniae* and NTHi in upper respiratory and otolaryngologic disease. While further investigation of the specific mechanisms that regulate community virulence are needed to better characterize the function of microbial communities in the context of polymicrobial disease, these studies may influence the development of new diagnostics and therapeutics. This will lead to more directed treatment of CRS and other chronic upper respiratory diseases worsened by the presence of *S. pneumoniae* and NTHi.
